# IL-38 promotes the development of prostate cancer

**DOI:** 10.3389/fimmu.2024.1384416

**Published:** 2024-05-08

**Authors:** Huiyan Wu, Jing Yang, Liuhong Yuan, Zhenyu Tan, Xiuqin Zhang, Brett D. Hambly, Shisan Bao, Kun Tao

**Affiliations:** ^1^ Department of Pathology, Tongji Hospital, Tongji University, Shanghai, China; ^2^ Department of Pathology, Tongren Hospital, Shanghai Jiaotong University School of Medicine, Shanghai, China

**Keywords:** prostate cancer, IL-38, PD-1, CD4, CD8, prognosis

## Abstract

**Introduction:**

Prostate Cancer (PCa) remains a significant concern in male cancer-related mortality. Tumour development is intricately regulated by the complex interactions between tumour cells and their microenvironment, making it essential to determine which is/are key factor(s) that influence the progression of PCa within the tumour microenvironment.

**Materials and methods:**

The current study utilised histopathology and immunohistochemistry to determine the expression of IL-38 in PCa and analysed the correlation between the expression level of IL-38 within PCa and clinical pathological characteristics.

**Results:**

There was a significant increase in IL-38 expression in PCa tissues compared to adjacent non-PCa tissues (P < 0.0001). In addition, IL-38 expression was significantly higher in tumour cells with a high proliferation index compared to those with a low value-added index. ROC curve analysis demonstrated that IL-38 has high specificity and sensitivity for the diagnosis of PCa (AUC=0.76). Moreover, we Probed the cellular source of IL-38 in prostate cancer tissue by immunofluorescence double staining. Additionally, within PCa, the expression of IL-38 was inversely correlated with the expression levels of CD8 and PD-1. Survival analysis revealed a significantly lower overall survival rate for PCa patients with high IL-38 expression (P=0.0069), and when IL-38 was co-expressed with CD8, the survival rate of the IL-38^high^/CD8^low^ group was decreased significantly. Multivariate analysis indicated that the expression level of IL-38 and TNM staging were independent predictors of survival in PCa patients.

**Conclusion:**

These findings suggest that IL-38 plays a crucial role in the development of PCa, and the exploration of the correlation between IL-38 and various immune factors in the tumour microenvironment further reveals its mechanism of action, making it a potential target for immunotherapy in PCa.

## Introduction

Prostate cancer (PCa) ranks as the second most prevalent cancer and the fifth leading cause of cancer-related mortality among men (1). It is estimated that annually there are approximately 1,414,259 new PCa cases and 375,304 related deaths globally, with rising incidence attributed to economic growth, an aging population, and lifestyle changes (2). The five-year survival rate for metastatic PCa falls below 30% (3), although localised tumours exhibit a curative response to radical prostatectomy and radiotherapy in about 70% of patients. However, early screening, primarily relying on prostate-specific antigen (PSA), poses the risk of clinical overtreatment (4). Consequently, there is a critical importance to identify a new biomarker with high specificity and sensitivity to facilitate early diagnosis and treatment.

Chronic inflammation and sustained immune responses play pivotal roles in PCa development and progression (5). PCa can evade the immune system through mechanisms such as down-regulation of HLA class I, inducing T-cell apoptosis, or secreting immunosuppressive cytokines like TGF-β, thereby increasing T regulatory cells (6). Tumorigenesis is intricately regulated by interactions between tumour cells and their microenvironment, and influencing cytokines to stimulate the immune system may lead to robust anti-tumour immunity (5, 7). Identifying cytokines affecting PCa patient survival not only holds prognostic value but also presents potential therapeutic targets by modulating the tumour microenvironment.

IL-38, a relatively obscure cytokine in the IL-1 family, has demonstrated extensive anti-inflammatory effects in numerous studies (8, 9). Dysregulation of IL-38 disrupts the balance between anti-inflammatory and pro-inflammatory microenvironments, triggering host immunity and contributing to the onset of various autoimmune diseases (9). We have demonstrated previously a significantly reduced IL-38 expression in colorectal cancer compared to surrounding non-colorectal cancer tissue, with higher levels of IL-38 correlating with tumour differentiation and serving as an independent prognostic marker (10). However, by contrast, upregulated IL-38 has been observed within lung non-small cell carcinoma (11), increased IL-38 expression was associated with lung tumour progression and poor prognosis. The difference between colorectal IL-38 and lung IL-38 expression may be due to the higher load of microflora within the colon compared to the lung, although both organs are classified as mucosal associated lymphoid organs. Subsequently, there may be different host immunological regulatory mechanism between the two organs during the development of malignancies.

Anti-PD-1/PD-L1 immune checkpoint inhibitor therapy has been quite extensively applied in the management of malignancies, since the role of these molecules has been well documented during the development of cancers (12), although some serious adverse responses can occur (13). However, the response of metastatic PCa to these drugs has been poor (14), likely due to PCa generally exhibiting a low tumour mutational burden (fewer tumour antigens), with a consequent low immune/inflammatory response.

Additional findings have revealed the role of IL-38 in promoting tumour growth by down-regulating CD8^+^ tumour infiltrating lymphocytes in the lung cancer tumour microenvironment (15, 16). However, the association of IL-38 with histological grade and survival in PCa remains unexplored.

In the current study, we employed histopathological and immunohistochemical methods to quantify IL-38 expression in PCa and analyse its correlation with clinicopathological features. Using immunofluorescence double staining, we explored the cellular origin of IL-38 in prostate cancer tissues and compared the differences in IL-38 expression in tumour glands with different proliferation indices. Cox proportional hazards regression analysis, both univariate and multivariate, demonstrated IL-38 as a specific and sensitive biomarker for predicting PCa patient survival. Our findings suggest that IL-38 may play a crucial role in PCa development. Furthermore, we investigated the correlation between IL-38 and the expression levels of CD4, CD8, CD20 and PD-1 in prostate tumour tissues, shedding light on the mechanisms through which IL-38 promotes PCa progression. These insights position IL-38 as a potential target for future immunotherapies in PCa.

## Materials and methods

### Collection of clinical pathological data and follow-up information

The current study identified 135 cases of prostate adenocarcinoma (acinar type) and 60 cases of benign prostatic hyperplasia, confirmed with histopathology at Tongren Hospital in Shanghai from 2017 to 2021. The median age of the patients was 71 years (range: 48-81 years). All cancer patients underwent the same radical prostatectomy without preoperative chemotherapy. BPH was performed as TURP, as described (17). Clinical pathological data were obtained from the electronic database, Tongren Hospital. The Gleason score, TNM staging, prognostic grouping (18) were re-evaluated for all 135 specimens. PCa tissues from each patient were matched with adjacent normal prostate tissues.

Follow-up data for PCa patients were obtained from the *Shanghai Changning District Centre for Disease Prevention and Control*, with follow-up conducted until February 2023. The final follow-up information was obtained for 116 patients, yielding a follow-up rate of 85.9%. After excluding cases with incomplete clinical pathological information, a total of 111 cases were included in the survival curve analysis, including 16 deaths and 100 survivors, with the longest survival time being 64 months.

The study has been approved by the Human Ethic Committee, Tongren Hospital, Shanghai Jiaotong University School of Medicine.

### Creation of tissue microarrays (TMAs)

To create TMAs, representative prostate cancer tissues and adjacent normal tissues were selected from HE stained slides. Tissue cores with a diameter of approximately 2 mm were extracted from the corresponding areas on the paraffin blocks. These tissue cores were systematically arranged and embedded into pre-made recipient paraffin blocks (Model: UB06-1, Yubei, Shanghai). The resulting TMAs were then sectioned at 4 µm. The morphology of the cancer tissues and adjacent non-cancer tissues on sequential sections on the array corresponded well with that of the originally selected regions on the HE slides.

Histopathology diagnosis followed the 2022 *World Health Organization* Classification of Tumours of the Prostate (19).

### Immunohistochemistry

The sections were immunohistochemically stained as described previously (10, 20). Briefly, the sections were treated with EDTA antigen retrieval solution (Beyotime, Shanghai), followed by endogenous peroxidase blocking for 15 minutes and blocking with foetal bovine serum (Ausbian Australia). Primary antibodies, all obtained from Abcam, Cambridge, UK, against human IL-38 (Ab180898), human CD4 (Ab133616), human CD8 (Ab237710), human CD20 (Ab78237), and human PD-1 (Ab237728) were incubated at room temperature for 1 hour. HRP-labelled mouse/rabbit universal secondary antibody polymer (Beijing Sequoia Jinqiao Biological Technology) was incubated for 30 min. The sections were visualised using DAB, counterstained with hematoxylin, for microscopic observation.

Digitalization of the slides was performed using the NANO Zoomer series digital scanner 2.0 (Hamamatsu, Japan). Quantification of IL-38, CD4, CD8, CD20, and PD-1 expression was carried out, using Halo digital imaging analysis software 2.0 (Indica Labs, USA). Each point of PCa tissue, stromal area, and blank region was manually outlined, excluding areas with tissue artifacts. Staining intensity thresholds were set, and cells with staining intensity exceeding the negative threshold were considered positive. The software utilised a predefined training algorithm to calculate the number and staining intensity of positive cells in tumour and stromal areas, providing the positive cell units per unit area.

### Immunofluorescent staining

To confirm the source of IL-38 from the prostate cancer and the relationship between IL-38 and cell proliferation indices, immunofluorescent double staining was applied, as described previously (21, 22). Briefly the sections were stained with anti-IL-38 and anti-P504S (RMA-0546, Fuzhou Maixin Biological Technology, China), anti-CD3, anti-CD68, anti-CD138, anti-CD20, and anti-Ki67 (ZM-0166, Beijing Sequoia Jinqiao Biological Technology), where IL-38 was used at a dilution of 1:400 (red fluorescence), anti-CD3, CD68, and CD138 were used at dilutions of 1:100, 1:1000, and 1:8000 respectively (green fluorescence), and anti-P504S and Ki67 were both used at a ready-to-use concentration (green fluorescence).

The numbers of IL-38^+^ cells were identified with containing CD138^+^ plasma cells, CD3^+^ pan-T cells, CD68^+^ macrophages, CD20^+^ B-lymphocyte, P504S^+^ prostate tumour epithelial cells or Ki67 proliferative marker. These double staining sections were quantified, using the HALO Image analysis platform, as described previously (22) (21). The percentage of the double staining was calculated from the randomly selected 5 visual fields from each section, and quantified using Imagepro Plus V7 (23). The quantification was performed under double-blind fashion to reduce/minimize bias.

### Statistics

Statistical analyses were conducted, using GraphPad Prism 8.0.1 and SPSS 26.0 software packages. The comparison between two paired groups was performed using the Wilcoxon signed-rank test. Unpaired group comparisons were conducted using the Mann-Whitney U test. Multiple group comparisons were assessed using the Kruskal-Wallis H test. ROC curve analysis was employed to evaluate the diagnostic specificity and sensitivity of IL-38 expression levels in cancer tissues and adjacent normal tissues for PCa. Spearman correlation coefficient analysis was utilised to examine the correlation between IL-38 expression in cancer tissues and the expression levels of CD4, CD8, CD20, and PD-1.

High and low expression groups of IL-38 within PCa were defined based on the median expression level of IL-38 in PCa tissues. Total survival duration was defined as the number of days from surgery to the last follow-up or death. Kaplan-Meier survival curves were constructed, and the impact of various immune markers on the survival rate of PCa patients was analysed using the log-rank test. The Cox proportional hazards model was employed to identify prognostic factors influencing survival. Results were considered statistically significant at P < 0.05.

## Results

### Demographics of PCa and BPH patients

Demographic data for 135 cases of primary prostate cancer and 60 cases of benign prostatic hyperplasia patients are shown in **Table 1**. No PCa patients received chemotherapy prior to surgery. Due to incomplete clinical data for a small number of patients and occasional loss of individual sites (for example, loss of non-cancer prostate tissue adjacent to cancer tissue) during the tissue microarray construction process, the actual number of samples with matching cancer and adjacent non-cancer tissue included in the analysis was slightly reduced and totalled 128 matched patients. According to the latest version of the prostate cancer Gleason grading system (24) and TNM staging criteria (18), the number of patients with PCa Gleason scores < 7, = 7, and > 7 were 29, 68, and 38, respectively. The distribution of patients across stages I, II, III, and IV were 18, 67, 40, and 10 cases, respectively.

**Table 1:** Clinicopathological characteristics of patients with PCa and BPH.

Characteristics	No. Pts with PCa (with matched adjacent non-cancer tissue)	No. Pts with BPH
Number of patients	135 (128)	60
Age, years		
< 60	11 (11)	6
≥ 60-< 70	47 (45)	21
≥ 70-< 80	74 (69)	30
≥ 80	3 (3)	3
PSA, ng/ml		
< 10	45 (41)	43
≥ 10-<20	48 (45)	14
≥ 20	42 (42)	3
Gleason score		
≤ 6	29 (27)	
7	68 (66)	
≥ 8	38 (35)	
Grade group		
1	29 (27)	
2	34 (32)	
3	34 (34)	
4	16 (14)	
5	22 (21)	
T stage		
T1	0	
T2	87 (80)	
T3	48 (48)	
T4	0	
N stage		
N0	125 (118)	
N1	10 (10)	
M stage		
M0	135 (128)	
M1	0	
TNM		
I	18 (18)	
II	67 (60)	
III	40 (40)	
IV	10 (10)	
Vascular invasion		
no	115 (108)	
yes	17 (17)	
unknown	3 (3)	
Nerve invasion		
no	46 (42)	
yes	87 (84)	
unknown	2 (2)	
Prognostic grouping		
I	15 (14)	
II	50 (45)	
III	57 (56)	
IV	13 (13)	

### Expression of IL-38 in PCa, non-PCa and BPH tissues

**Figure 1:**
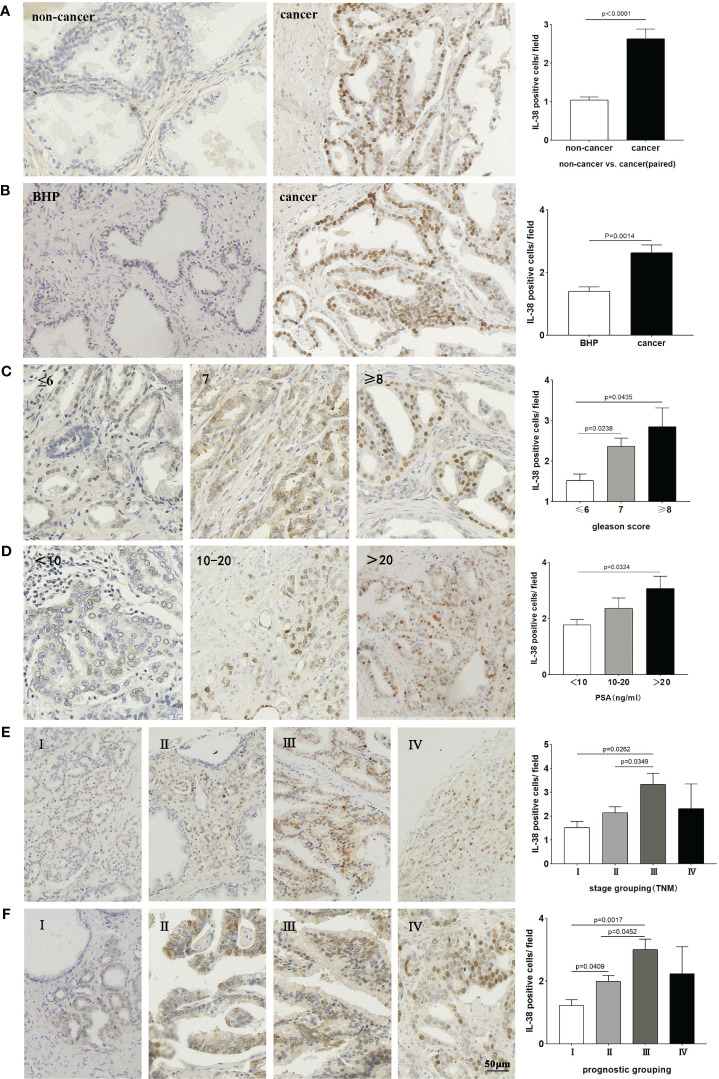
Compares the expression of IL-38 in paired cancer and non-cancer tissues, as well as, benign prostatic hyperplasia specimens, using the Wilcoxon signed-rank test **(A, B)**. The expression of IL-38 was compared between different Gleason scores **(C)**, serum PSA levels **(D)**, TNM staging **(E)**, and prognostic groups **(F)** using the Mann-Whitney U test. Representative images for each group are depicted in the photomicrographs. The original magnification is ×200.

Local IL-38 was predominantly expressed in the nuclei of tumour cells within PCa adenomatous glands, with a small amount of expression in the cytoplasm. There was significantly elevated IL-38 expression in PCa tissues, compared to that of non-PCa tissues BPH and (**Figures 1A, B**, P < 0.0001;P=0.0014, respectively). Additionally, there was a correlation between the Gleason score of PCa tissues and IL-38 (**Figure 1C**, P = 0.0435) or serum PSA and IL-38 (**Figure 1D**, P = 0.0324).

Furthermore, the PCa patients in TNM stage III exhibited significantly higher IL-38 expression than those in stages I and II (**Figure 1E**, P = 0.0262; P=0.0349), Similarly, the PCa patients in prognostic group III showed significantly higher IL-38 expression than those in groups I and II (**Figure 1F**, P = 0.0017; P=0.0452). It is noteworthy that the expression of IL-38 decreases in patients within the TNM stage IV group and prognostic group IV.

### ROC curve analysis of IL-38 in PCa cancer tissues and adjacent normal tissues

**Figure 2:**
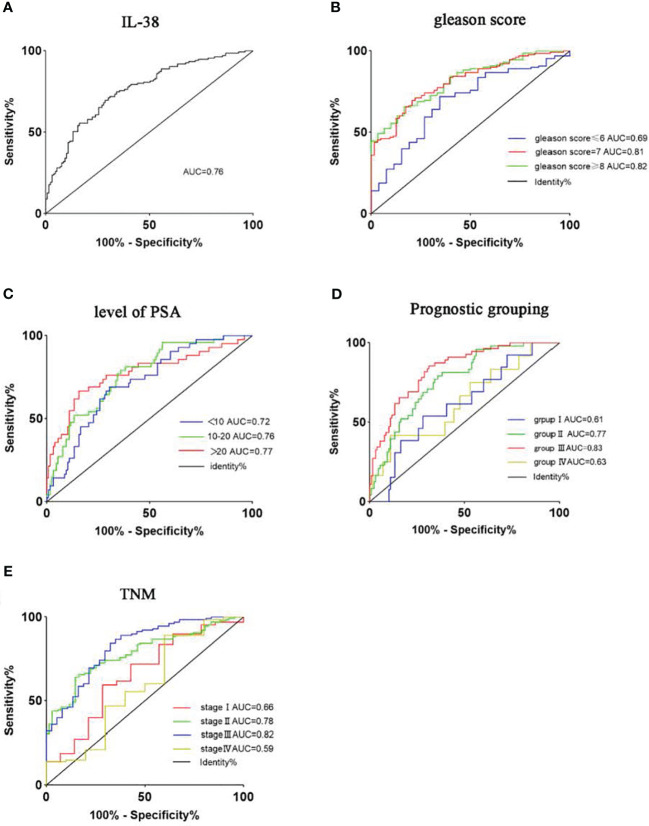
Utilises ROC curves to assess the sensitivity and specificity of IL-38 expression levels in diagnosing PCa. The area under the curve (AUC) for IL-38 is shown as follows: IL-38 **(A)**: AUC=0.76. Subgroup analyses were performed based on Gleason score **(B)**, serum PSA levels **(C)**, prognostic groups **(D)**, and TNM staging **(E)**.

To assess the specificity and sensitivity of IL-38 expression levels in diagnosing PCa, ROC curve analysis was conducted as described (20). We found that the area under the ROC curve (AUC) for IL-38 expression in PCa and adjacent non-PCa tissues was 0.76 (**Figure 2A**), suggesting a high specificity and sensitivity of IL-38 in distinguishing PCa from non-PCa tissues. As the Gleason score of cancer tissues increased (**Figure 2B**) and serum PSA levels increased (**Figure 2C**), the AUC of the ROC curve gradually increased, suggesting that IL-38 also has high specificity and sensitivity for distinguishing different Gleason scores and serum PSA levels in PCa. With the increase in TNM staging (**Figure 2E**) and prognostic grouping (**Figure 2D**), the AUC of the ROC curve also gradually increased. However, in TNM stage 4 and prognostic group 4, the AUC decreased. Due to the limited number of samples available for analysis (n=10; n=13) in stage 4, the AUC data for late-stage PCa exhibited larger fluctuations.

### Correlation between IL-38 and the expression of CD4, CD8, CD20, and PD-1 in PCa

**Figure 3:**
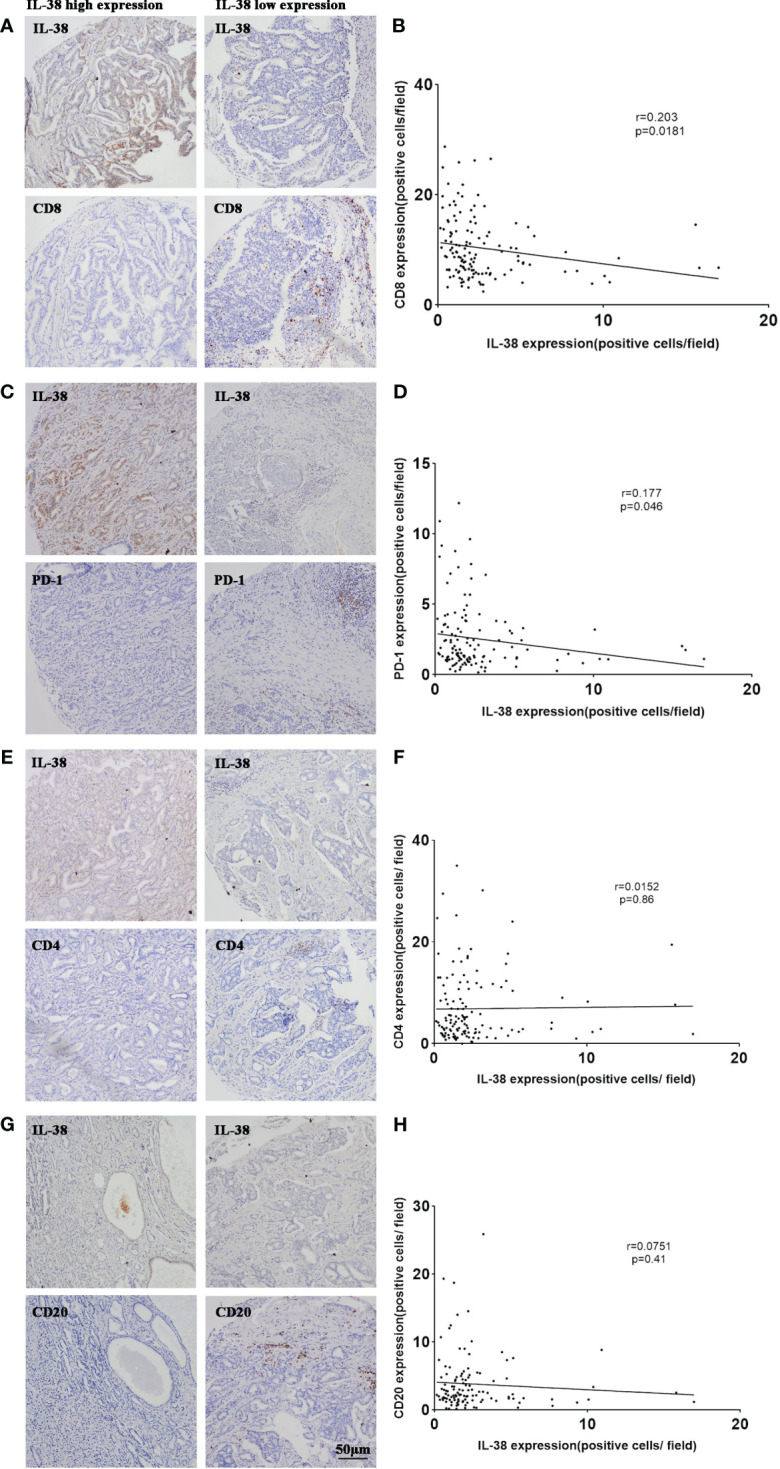
Correlation of IL-38 with the expression of CD4, CD8, CD20, and PD-1 in PCa. Correlation analyses were conducted to examine the relationship between IL-38 and the expression of CD4, CD8, CD20, and PD-1 in tumour tissues of prostate cancer patients. Representative images for each correlation are displayed in the microscopic photographs: IL-38/CD8 **(A)**, IL-38/PD-1 **(C)**, IL-38/CD4 **(E)**, IL-38/CD20 **(G)**. The correlation between IL-38 expression (X axis) with CD8 **(B)**, PD-1 **(D)**, CD4 **(F)**, or CD20 **(H)** expression (Y-axis) is presented. The original magnification is ×200.

To investigate the relationship between IL-38 and CD4^+^, CD8^+^ T lymphocytes in the tumour immune microenvironment of PCa, the correlation between IL-38 and the expression levels of CD4 and CD8 in PCa was analysed. Immunohistochemical quantification revealed that in PCa, the expression of IL-38 was negatively correlated with the expression of CD8 (r=0.203, p=0.0181, **Figures 3A, B**), but exhibited no correlation with CD4 expression (r=0.152, p=0.86, **Figures 3E, F**). Additionally, IL-38 showed an inverse correlation with PD-1 expression levels (r=0.177, p=0.046, **Figures 3C, D**), while no significant correlation was observed between IL-38 and CD20 expression (r=0.0751, p=0.41, **Figures 3G, H**).

### Identification of IL-38 source

**Figure 4:**
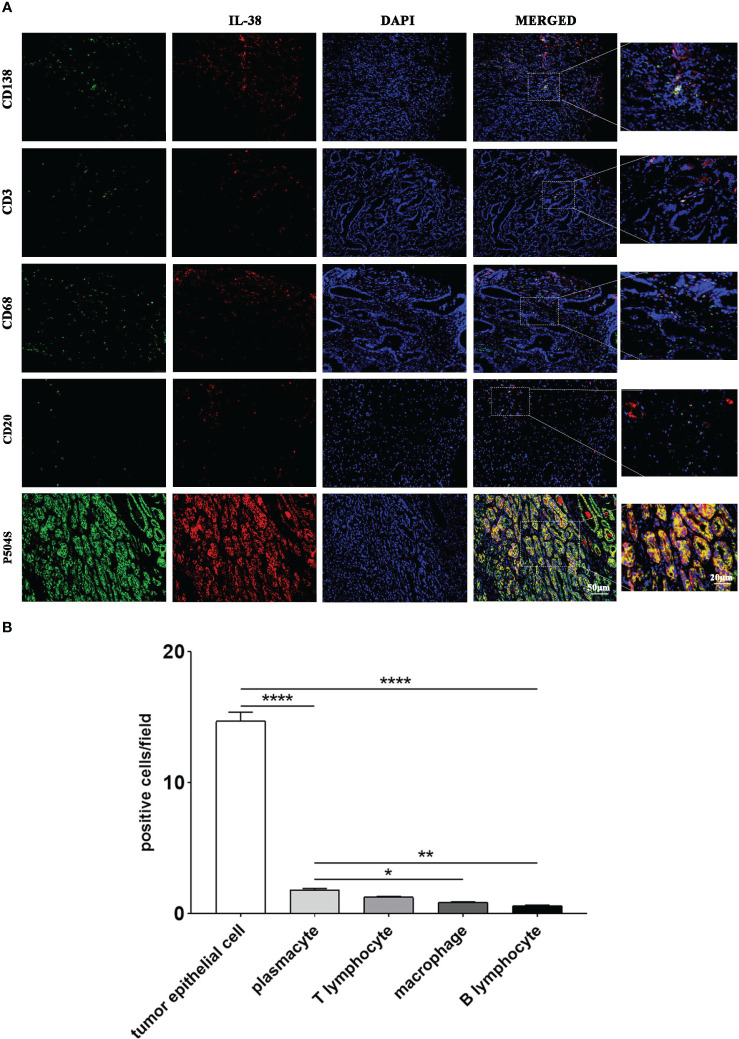
The cellular origin of IL-38 within prostate cancer tumour tissues was explored using immunofluorescence double staining. **(A)** Images of immunofluorescence double staining of IL-38 (red fluorescence) with P504S, CD138, CD3, CD68, and CD20 (green fluorescence); the original magnification x 200, and the magnified images were taken at 40x. **(B)** Quantitative analysis results of co-staining of each immune cell with IL-38. *, **, ***, **** represents p<0.05, 0.01, 0.001 or 0.0001.

In order to investigate the source of IL-38 and its localisation with each immune cell in prostate cancer tumour tissues, immunofluorescence double-staining of IL-38 with tumorigenic epithelial cells (P504S^+^) with each immune cell (CD138^+^ plasmacytes, CD3^+^ T cell, CD68^+^ macrophages, and CD20^+^ B cell) was performed. Representative images and quantitative analysis results are shown in **Figure 4**, showing that the number of IL-38/P504S^+^ tumorigenic epithelial cells was 14.73 per field. Then on the order of IL-38^+^/CD138^+^ plasmacytes, IL-38^+^/CD3^+^ T cell, IL-38^+^/CD68^+^ macrophages, and IL-38^+^/CD20^+^ B cells, which were 1.80, 1.27, 0.88 and 0.61 per filed in prostate cancer tumour and mesenchyme.

### IL-38 expression in cancer cells with different proliferation indexes

**Figure 5:**
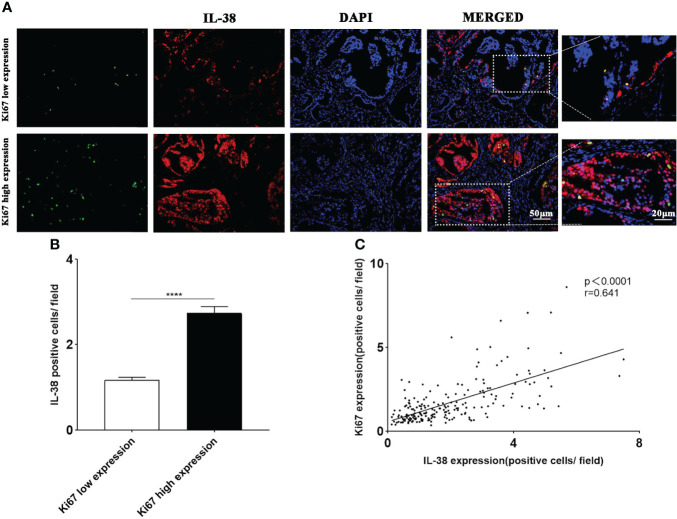
The expression of IL-38 in prostate tumour cells with different proliferation indices was explored using immunofluorescence double staining. **(A)** Images of immunofluorescence double staining of IL-38 (red fluorescence) with Ki67 (green fluorescence); original magnification ×200, magnified image taken at 40x magnification. **(B)** The expression of IL-38 was compared between tumour cells with different proliferation indices. **(C)** Correlation analyses were conducted to examine the relationship between IL-38 and the expression of Ki67 in tumour tissues of prostate cancer patients. **** represents p<0.0001.

To explore the differences in IL-38 expression in tumour cells with different proliferation indices, we co-stained IL-38 with Ki67 (proliferation marker) Representative immunofluorescence images are shown in **Figure 5A**. The expression of IL-38 was more than 2-fold higher in the Ki67^high^ group, compared to that in the Ki67^low^ (**Figure 5B**, P < 0.0001). There was significant correlation between IL-38 and Ki67 in the prostate cancer (**Figure 5C**, r = 0.641, P < 0.0001).

### Survival curve analysis of IL-38 expression levels stratified by CD8 and PD-1 expression in PCa tissues

The follow-up for the patient cohort was conducted until February 2023, with 116/135 (85.9%) PCa patients available for follow-up. Among the 116 patients, 16 had died, with the longest survival period being 64 months. Using the log-rank test, IL-38 expression levels were arranged, and the median was used as the cut-off value (distinguishing high and low expression groups of IL-38). The relationship between IL-38 expression levels and postoperative overall survival rate in PCa patients was then evaluated.

**Figure 6:**
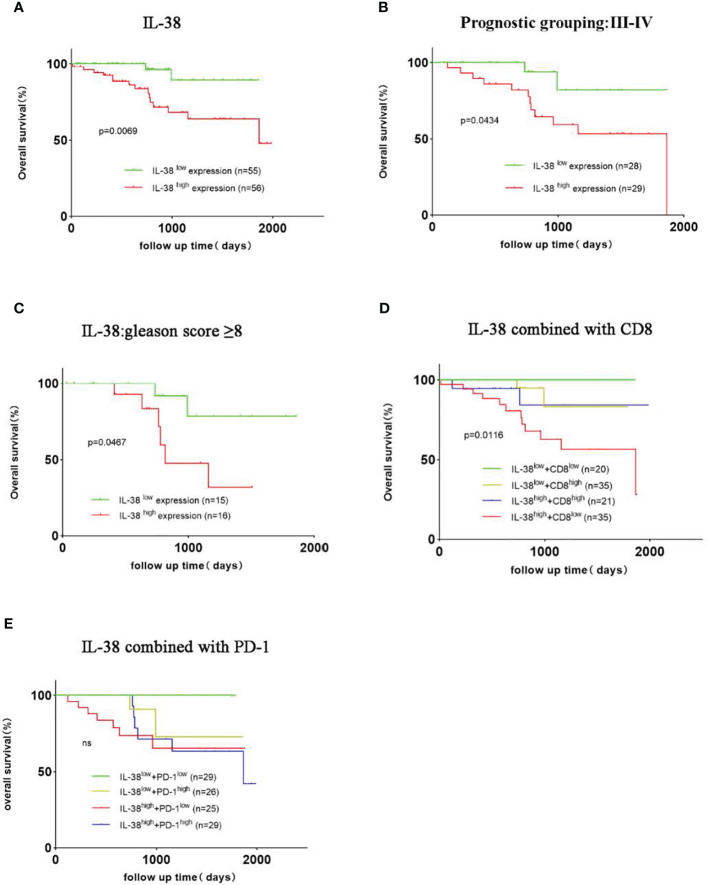
Survival Curves for PCa with high or low expression of IL-38, CD8, and PD-1. The Kaplan-Meier survival curves depicted the correlation between IL-38, CD8, PD-1 expression levels, and overall survival rates in prostate cancer patients. **(A)** Illustrates the correlation between IL-38 expression levels and overall survival in prostate cancer patients. Subgroup analyses were conducted for patients with prognostic groups III-IV **(B)** and Gleason score ≥ 8 **(C)**. Statistical analysis of the overall survival rates based on the combined expression of IL-38 and CD8 **(D)** and the combined expression of IL-38 and PD-1 **(E)** were performed using the LOG-RANK test.

The analysis revealed that PCa patients with low IL-38 expression had a significantly higher overall survival rate than those with high IL-38 expression (P=0.0069; **Figure 6A**). Further stratifying the PCa patients into two subgroups, namely the prognostic groups III-IV group (n=57) and Gleason score ≥ 8 group (n=31), demonstrated that in both the prognostic groups III-IV group (P=0.0434; **Figure 6B**) and Gleason score ≥ 8 group (P=0.0467; **Figure 6C**), patients with low IL-38 expression had significantly higher survival rates than those with high expression.

Dividing patients into four subgroups based on the joint expression of IL-38 and CD8 (4 groups: double high, double low, IL-38^high^/CD8^low^, IL-38^low^/CD8^high^), the results showed that patients in the IL-38^high^/CD8^low^ group (n=35) had a significantly lower overall survival rate than the other three groups, with significant differences observed among all groups (P=0.0116; **Figure 6D**). When considering the combined expression of IL-38 and PD-1, patients in the IL-38^high^/PD-1^high^ group exhibited a trend towards a lower overall survival rate than the other three groups, although the overall survival rates among the groups did not show significant differences (**Figure 6E**).

### Univariate and multivariate Cox analysis: the relationship between IL-38 expression in PCa tissues, CD8, PD-1, and various clinicopathological characteristics with patient survival time

A series of factors influencing the survival of PCa patients were subjected to both univariate and multivariate Cox analyses (**Table 2**). In the univariate analysis, IL-38 expression (HR 5.57; 95%CI 1.26-24.69; p=0.024), level of PSA (HR 1.98; 95%CI 1.04-3.77; p=0.038), T staging (HR 5.13; 95%CI 1.63-16.13; p=0.005), TNM staging (HR 2.56; 95%CI 1.45-4.54; p=0.001), and prognostic staging (HR 1.92;95%CI 1.03-3.5; p=0.041 emerged as important factors affecting the survival of prostate cancer patients. Multivariate analysis further revealed that IL-38 expression (HR 6.73; 95%1.36-33.19; p=0.019) and TNM staging (HR 14.03; 95%1.67-118.23; p=0.015) were independent and reliable biomarkers predicting the survival rates of these prostate cancer patients. However, other factors such as age, serum PSA levels, risk stratification for recurrence, prognostic grouping, lymph node metastasis, expression levels of CD8 and PD-1, and the combined expression of IL-38 with CD8 or PD-1 did not exhibit statistical significance in predicting the survival rates of prostate cancer patients.

**Table 2:** Univariate and multivariate analyses of the relationship between survival of PCa patients and IL-38, CD8 and PD-1.

Characteristics	Univariate analysis		Multivariate analysis	
	HR (95%CI)	P value	HR (95%CI)	P value
IL-38				
High/low	5.57 (1.26-24.69)	0.024	6.73 (1.36-33.19)	0.019
Age (years)				
<60/60-70/>70	1.84 (0.82-4.15)	NS		
PSA (ng/ml)				
<10/10-20/>20	1.98 (1.04-3.77)	0.038	1.24 (0.48-3.25)	NS
Gleason score				
≤6/7/≥8	2.00 (0.94-4.25)	NS		
Grade group				
1/2/3/4/5	1.45 (0.99-2.13)	NS		
T stage				
T1/T2/T3/T4	5.13 (1.63-16.13)	0.005	1.60 (0.39-6.52)	NS
Lymph node metastasis				
no/yes	0.83 (0.11-6.30)	NS		
TNM				
I/II/III/IV	2.56 (1.45-4.54)	0.001	14.03 (1.67-118.23)	0.015
Prognostic grouping				
I/II/III/IV	1.92 (1.03-3.59)	0.041	0.15 (0.02-1.12)	NS
Vascular invasion				
no/yes	2.64 (0.73-9.53)	NS		
Nerve invasion				
No/yes	1.41 (0.45-4.43)	NS		
CD8 High/low	0.33 (0.11-1.04)	NS		
PD-1 High/low	0.74 (0.26-2.12)	NS		
IL-38 and PD-1 DN/SP1/SP2/DP	1.58 (0.95-2.60)	NS		
IL-38 and CD8 DN/SP1/SP2/DP	1.55 (0.89-2.71)	NS		

DP, double high; DN, double low; SP1, IL-38^low^+CD8^high^/IL-38^low^+PD-1^high^; SP2, IL-38^high^+CD8^low^/IL-38^high^+PD-1^low^.

NS, no statistical significant.

## Discussion

In this study, elevated levels of IL-38 in PCa tissues were identified compared to BPH and matched non-PCa samples (1). A positive correlation was found between IL-38 and Gleason score, PSA concentration, and PCa differentiation. Additionally, a correlation with AJCC stages was noted, except for stage IV (1). Prostatic IL-38 exhibited an inverse correlation with infiltrating CD8^+^ or PD-1, but not with CD4^+^ cells or CD20^+^ B cells (1). Furthermore, PCa patients with high IL-38 expression demonstrated significantly poorer 5-year survival rates compared to those with low IL-38 expression, consistent with Gleason score but not with PD-1 expression. Notably, IL-38, particularly in TNM stage IV PCa patients, emerged as a reliable prognostic biomarker through multivariate analysis.

The significantly elevated level of prostate IL-38 in PCa compared to BPH and non-PCa samples implies a potential role for IL-38 in PCa development. Although the exact function of IL-38 in PCa development remains unclear, we hypothesise that increased expression of IL-38 may represent an attempt to mitigate inflammation in the microenvironment. This speculation is grounded in the understanding that malignancy tends to exist within a pro-inflammatory microenvironment, especially in advanced PCa tissues exhibiting necrosis and/or apoptosis. However, the anti-inflammatory role of IL-38 may be compromised in susceptible individuals through downstream signalling pathways, such as IL-36r (25). Notably, there are three subunits of IL-36 with various differential roles in the development of cancer, as supported by our previous findings in colorectal cancer (26).

The high expression of IL-38 in PCa may contribute to the downregulation of host immunity against PCa, promoting its progression. This aligns with our current finding of a correlation between IL-38 and PSA, as well as Gleason score. Interestingly, IL-38 levels were higher in BPH than in non-cancerous tissues, consistent with the expression of IL-38 in lung small cell carcinoma, suggesting a potential role in enhancing tumour development (11). The prostate is generally aseptic under normal conditions, but BPH and/or prostatitis may not always be aseptic, and it may induce inflammation.

The positive correlation between prostate IL-38 levels and Gleason score in PCa tissues underscores the potential involvement of IL-38 in prostate cancer tumorigenesis. The role of inflammation in cancer development is complex, with acute inflammation appearing protective, while chronic inflammation contributes to carcinogenesis (27).

From the identified source of IL-38^+^ cells, the highest was prostate epithelial cells, supporting the notion that IL-38 promotes the development and/or progression of prostate cancer. Our hypothesis is supported by the finding in lung small cell carcinoma (11). The exploration of the source of leucocytes demonstrated the order of plasmacytes, T cells, macrophages, B cells. This finding suggests that there is involvement of leucocytes during the development of prostate cancer. However the precise underlying mechanism of these leucocytes in the pathogenesis reminds to be explored. Additionally, such finding might suggest the potential therapeutic target for precision medicine.

Interestingly the finding from prostate cancer is differ from these of colorectal cancer (10), showing IL-38 is protective during the development of colorectal cancer. The discrepancy between prostate cancer and colorectal cancer may be due to different microenvironment, probably mainly due to flora in the regions, i.e. almost aseptic in prostate cancer, but huge micro-organisms in the colorectal area.

Our data demonstrated that IL-38 was positively correlated with the expression of Ki67 in PCa tissues, which is consistent with that IL-38^high^ was more than 2-fold higher in Ki67 than IL-38^low^ group. Such finding was further supporting our speculation that IL-38 promotes tumour development in prostate cancer. This is in line with others, showing that Ki67 correlates with the prognosis of a variety of tumours (28), and Ki67^high^ expression in prostate cancer patients shows poor prognosis (29).

An inverse correlation was observed between IL-38 expression and the infiltration of cytotoxic CD8^+^ cells in PCa tissue, providing additional evidence for the immune-inhibiting role of IL-38 in the microenvironment. This suggests that IL-38 suppresses host immunity against PCa development, aligning with other studies demonstrating that intertumoral CD8^+^ T-cell infiltration is associated with improved survival in PCa patients (30). The role of CD8^+^ T cells in PCa development warrants further exploration in future studies.

Furthermore, there was an inverse correlation between IL-38 and PD-1 expression in PCa tissues. Given the efficacy of anti-PD-1 therapy in malignancy management, our findings regarding IL-38 in PCa confirm its potential role in enhancing PCa development *in vivo*. Future investigations should explore whether there is a synergistic role between IL-38 and PD-1 in PCa, necessitating larger sample sizes and multi-centre studies for a comprehensive understanding.

Surprisingly, no correlation was observed between IL-38 and infiltrating CD4^+^ Th cells or CD20^+^ B cells in PCa, suggesting that neither CD4^+^ Th cells nor CD20^+^ B cells are directly involved in PCa development, and/or that the local IL-38 expression may be too potent, quenching the functions of these cells in the PCa microenvironment. The presence of CD4^+^/CD25^+^ regulatory T cells, known to be significantly higher in PCa tissues, indicates an immunosuppressive role in PCa development, requiring further investigation (31).

Additionally, there was no significant correlation between IL-38 and CD20^+^ B cells in PCa, suggesting that humoral immunity may not be efficiently involved in PCa. Nonetheless, substantial infiltrating CD20^+^ B cells in PCa have been reported as a potential therapeutic target (32). The discrepancy between our findings and those of other studies is unclear, but may be attributed to racial differences, impacting infiltrating leukocyte phenotypes and therapeutic outcomes.

Moreover, a highly significant difference was observed between IL-38^low^ and IL-38^high^ expression in PCa, providing strong support for the notion that IL-38 enhances PCa development and represents a potential therapeutic target. This observation aligns with findings in non-small cell lung cancer but contrasts with colorectal cancers (33), likely influenced by organ-specific factors impacting host immunity during malignancy development (11).

Consistency in IL-38 expression is evident among high-risk recurrence PCa patients, particularly pronounced in prostate tissues from PCa patients with Gleason scores > 8, indicating a more severe malignancy. These data imply that IL-38 is crucial in this susceptible cohort. The combination of IL-38 expression and CD8^+^ infiltrating T cells in PCa plays a critical role in determining survival, supporting the synergistic role of IL-38 and CD8^+^ infiltration, where IL-38 enhances PCa development by inhibiting CD8^+^ killer T cells in PCa tissues. Conversely, the combination of IL-38 and PD-1 shows almost no impact, suggesting that PD-1 may not participate substantially in the development of PCa, consistent with other findings (9). However we acknowledge that the sample size was relatively small, there were ~20 samples in each group after stratification. Thus the data obtained might not be the most reliable which require further investigation by increasing sample size as well as from multiple centres in future.

Limitation: We speculate that the observed decline in IL-38 expression in late-stage patients may be attributed to two factors: First, Relative limited sample size, necessitating further expansion for confirmation, especially in advanced prostate cancer patients; second, late-stage patients often exhibit cellular necrosis and apoptosis in tumour tissues, which may affect IL-38 secretion, leading to a downward trend in IL-38 expression.

Interestingly, the information from TCGA data shows there is little IL-38 mRNA in PCa. There are two differences between ours and the TCGA data: it shows mRNA only, and the samples are from the different regions perhaps with different genetical backgrounds. Such differences will be explored in future by multi-centre study.

Although there is an inverse correlation between IL-38 and PD-1, suggesting IL-38 might enhance the progression of prostate cancer. However the survival curve isn’t highly convinced enough, as well as relatively low r value, which might be due to relative small in sample size and/or other interaction(s) with the host immunity. The precise mechanism will be explored in future.

In summary, our study might provide some useful information for the potential prognostic and immunomodulatory roles of IL-38 in PCa, laying the groundwork for further exploration and therapeutic considerations in the management of prostate cancer.

## Data availability statement

The raw data supporting the conclusions of this article will be made available by the authors, without undue reservation.

## Ethics statement

The studies involving humans were approved by Tongren Hospital, Shanghai Jiaotong University School of Medicine. The studies were conducted in accordance with the local legislation and institutional requirements. The participants provided their written informed consent to participate in this study. Written informed consent was obtained from the individual(s) for the publication of any potentially identifiable images or data included in this article.

## Author contributions

HW: Writing – original draft, Software, Methodology, Investigation. JY: Writing – review & editing, Supervision, Methodology, Data curation. LY: Writing – review & editing, Software, Investigation. ZT: Writing – review & editing. XZ: Writing – review & editing, Methodology. BH: Formal Analysis, Writing – review & editing. SB: Conceptualization, Writing – review & editing. KT: Writing – review & editing, Supervision, Funding acquisition, Conceptualization.
